# Pembrolizumab for a patient with metastatic castration‐resistant prostate cancer with microsatellite instability‐high

**DOI:** 10.1002/iju5.12144

**Published:** 2020-02-14

**Authors:** Motohiro Fujiwara, Yoshinobu Komai, Takeshi Yuasa, Noboru Numao, Shinya Yamamoto, Iwao Fukui, Junji Yonese

**Affiliations:** ^1^ Department of Urology Cancer Institute Hospital Japanese Foundation for Cancer Research Tokyo Japan

**Keywords:** microsatellite instability, pembrolizumab, prostate cancer

## Abstract

**Introduction:**

We report the case of a patient with metastatic castration‐resistant prostate cancer with microsatellite instability‐high who was treated with pembrolizumab after cabazitaxel administration.

**Case presentation:**

A 58‐year‐old patient with heavily pretreated metastatic castration‐resistant prostate cancer, whose prostate surgical specimen was disclosed as microsatellite instability‐high, underwent pembrolizumab therapy. After initiation of pembrolizumab, his prostate‐specific antigen level decreased, imaging findings showed good response with lymph node shrinkage, and his walking difficulty decreased dramatically.

**Conclusion:**

The rarity of microsatellite instability‐high tumor in castration‐resistant prostate cancer may hamper pembrolizumab administration. This potentially active agent should be considered as part of a treatment regimen for patients with microsatellite instability‐high castration‐resistant prostate cancer. To the best of our knowledge, this is the first report of a Japanese castration‐resistant prostate cancer patient who demonstrated clinical benefit from pembrolizumab treatment.

**Abbreviations & Acronyms**
ADT: androgen deprivation therapy
CRPC: castration‐resistant prostate cancer
CT: computed tomography
MMR: mismatch repair
MSI: microsatellite instability
MSKCC: Memorial Sloan Kettering Cancer Center
ProGRP: pro‐gastrin‐releasing peptide
PSA: prostate‐specific antigen
PS: performance status
QOL: quality of life

**Keynote message**

We report the case of a patient with metastatic CRPC with MSI‐high treated with pembrolizumab after cabazitaxel administration. The rarity of MSI‐high tumor in CRPC may hamper pembrolizumab administration. This potentially active agent should be considered in treatment of these patients.

## Introduction

For patients with metastatic CRPC, various treatment agents, including abiraterone, cabazitaxel, docetaxel, enzalutamide, and radium‐223, have been approved in Japan.^1^, ^2^, ^3^, ^4^, ^5^ However, there are patients who have already received all of these agents and need a different systemic therapy.

On December 25, 2018, pembrolizumab was approved for the treatment of metastatic solid tumors in patients with MSI‐high in Japan.^6^, ^7^ Despite coverage under the Japanese universal health insurance system for pembrolizumab in MSI‐high tumors regardless of the tissue origin, the efficacy and safety profile of pembrolizumab for the CRPC patients with MSI‐high are poorly documented. We report the case of a patient with metastatic CRPC, who underwent pembrolizumab after progression despite various systemic therapies.

## Case presentation

**Figure 1:**
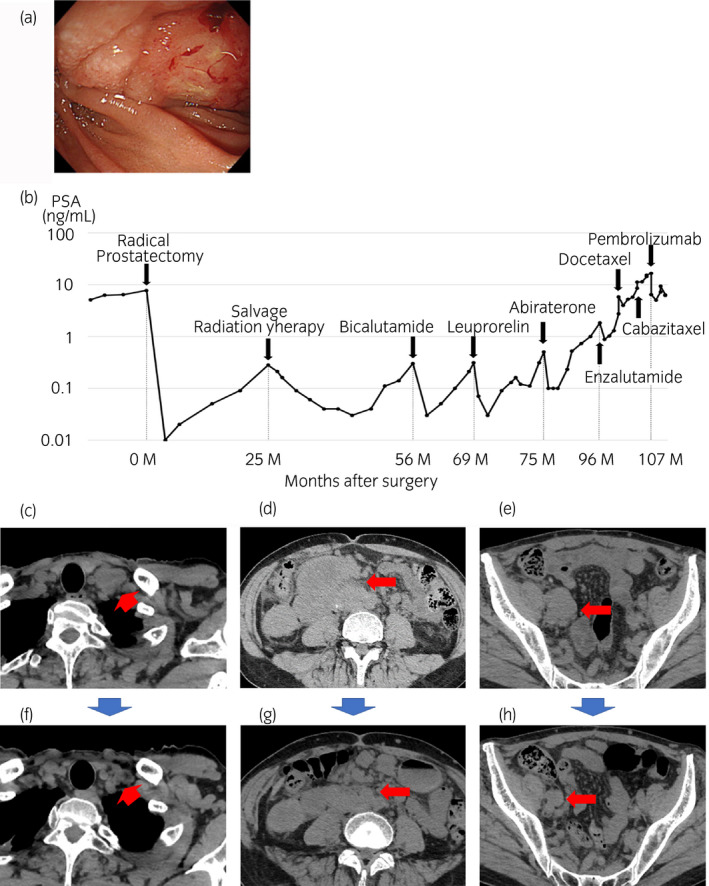
Clinical course of a metastatic CRPC patient with MSI‐high treated with pembrolizumab. (a) Endoscopic appearance of the duodenal invasion of the retroperitoneal lymph node. (b) Schematic clinical course with the serum PSA level. Thoracoabdominal CT appearance of (c) cervical, (d) retroperitoneal, and (e) pelvic lymph node metastases before pembrolizumab administration. Thoracoabdominal CT appearance of (f) cervical, (g) retroperitoneal, and (h) pelvic lymph node metastases after pembrolizumab administration.

A 52‐year‐old man, who has no medical history and no family history of prostate cancer, was diagnosed with prostate cancer (initial serum PSA level, 7.73 ng/mL; Gleason score, 4 + 4; cT2aN0M0). He underwent radical prostatectomy and his PSA level decreased to 0.00 ng/mL. However, his PSA level gradually increased to 0.28 ng/mL at 25 months after surgery. CT and bone scintigraphy showed no metastasis. He underwent salvage radiation therapy (70 Gy, 35 fraction) to the pelvic floor and his PSA level decreased to 0.04 ng/mL. However, his PSA level gradually increased to 0.30 ng/mL at 5 years after surgery. CT revealed right internal iliac lymph node metastasis. He started ADT with bicalutamide. After starting ADT, his PSA level was well controlled below 0.10 ng/mL for 23 months. However, his PSA level increased to 0.50 ng/mL at a castrated serum testosterone level of <0.3 ng/dL. Despite sequential treatment with abiraterone acetate (12 months), enzalutamide (4 months), docetaxel (three cycles), and cabazitaxel (four cycles), his PSA level increased to 16.6 ng/mL. Serum level of ProGRP level was within normal range (56.1 pg/mL, normal range 0–71.9 pg/mL) and reevaluation of primary prostate biopsy disclosed that there is no subset of neuroendocrine differentiation. CT demonstrated enlarged cervical, retroperitoneal, and pelvic lymph node metastases. The enlarged retroperitoneal lymph nodes invaded the duodenum and caused gastrointestinal bleeding (Fig. 1a). Additionally, he showed gait disturbance because of the enlarged retroperitoneal lymph node and his QOL and PS had worsened (PS, 2–3). A schematic of his clinical course is shown in Figure 1b. The enlarged cervical, retroperitoneal, and pelvic lymph node metastases are shown in Figure 1c–e, respectively.

During this treatment period, in December 2018 in Japan, pembrolizumab was approved for the patients with MSI‐high tumors. The patient's prostate surgical specimen showed MSI‐high prostate cancer. In June 2019, he started on pembrolizumab infusion intravenously once every 3 weeks per cycle as a standard protocol. Following five cycles of pembrolizumab therapy, his PSA level decreased 63% (16.6–6.1 ng/mL) and CT demonstrated shrinkage of the metastatic cervical, abdominal, and pelvic lymph nodes (>30%, Fig. 1f–h). Additionally, his walking difficulty decreased and his PS improved (PS: 1). The patient is continuing treatment with pembrolizumab.

## Discussion

The DNA MMR system plays key roles in repairing mismatched nucleotides that are caused by external physical or chemical damage.^8^ Inactivation of MMR genes caused by a germline and/or somatic hypermutation alterations induces high frequency of MSI.

We experienced heavily pretreated metastatic CRPC in patients with MSI‐high tumors who are treated with pembrolizumab. Since the approval, we have examined MSI‐high test for 10 metastatic CRPC patients, but this patient is the only patient who demonstrated positive MSI‐high test results (1/10, 10%). MSI‐high prostate cancer is uncommon, and its rarity seems to hamper the spread of immune checkpoint inhibitor therapy for CRPC. Abida *et al*.^9^ reported the prevalence of MSI‐high in patients with prostate cancer at the MSKCC. In their report, 1346 patients underwent paired tumor and germline sequencing, and only 32 of 1033 (3.1%) had MSI‐high or MMR‐deficient disease, among whom seven (21.9%) carried a germline mutation in the MMR genes.^9^ Among these patients, six patients had more than one tumor analyzed, and two of these patients (33.3%) showed an acquired MSI‐high phenotype later in their disease course.^9^ When an archive specimen did not show MSI‐high status, we may need to obtain fresh tissues from the metastatic sites.

There have been several reports on the aggressive pathology of MSI‐high prostate cancer. Guedes *et al*.^10^ reported that 1.2% (14/1176) had MSH2 loss in prostate cancer. Among these patients, 8% (7/91) of adenocarcinomas with a Gleason pattern 5 (Gleason score, 9–10) had MSH2 loss. Additionally, two of 43 patients (5%) with neuroendocrine prostate cancer had MSH2 loss.^10^ Schweizer *et al*.^11^ reported that four of 10 (40%) ductal cancer patients had MMR gene alteration (*n* = 2, *MSH2*; *n* = 1, *MSH6*; and *n* = 1, *MLH1*). Thus, MSI‐high testing could be proactively performed in CRPC patients with a primary Gleason score of 5, neuroendocrine histology, and ductal adenocarcinoma.

Regarding the efficacy of immune checkpoint inhibitors for prostate cancer, early clinical trial disclosed that none of the 17 CRPC patients demonstrated objective responses.^12^ Tucker *et al*. reported 25 cases of CRPC patients who were treated with pembrolizumab and one of these patients had MSI‐high. His PSA level showed a ≥90% decline.^13^ Thus, the efficacy of immune checkpoint inhibitors for the CRPC patients with MSI‐high is poorly documented. Pembrolizumab for CRPC requires further investigation in clinical practice.

In conclusion, treatment of these CRPC patients with pembrolizumab is poorly documented. The rarity of MSI‐high tumor in CRPC may hamper pembrolizumab administration. This potentially active agent should be considered in treatment of these patients. This is the first report in a Japanese CRPC patient who demonstrated a clinical benefit from pembrolizumab.

## Conflict of interest

T. Yuasa received remuneration for a lecture from MSD (Tokyo, Japan). The other authors declared no conflict of interest.
